# Relationship of literacy and heart failure in adults with diabetes

**DOI:** 10.1186/1472-6963-7-98

**Published:** 2007-07-02

**Authors:** Ann S Laramee, Nancy Morris, Benjamin Littenberg

**Affiliations:** 1 Fletcher Allen Health Care, McClure 1 Cardiology, Burlington, Vermont USA; 2 College of Nursing and Health Sciences, University of Vermont, Burlington, Vermont USA; 3 College of Medicine, University of Vermont, Burlington, Vermont USA

## Abstract

**Background:**

Although reading ability may impact educational strategies and management of heart failure (HF), the prevalence of limited literacy in patients with HF is unknown.

**Methods:**

Subjects were drawn from the Vermont Diabetes Information System Field Survey, a cross-sectional study of adults with diabetes in primary care. Participants' self-reported characteristics were subjected to logistic regression to estimate the association of heart failure and literacy while controlling for social and economic factors. The Short Test of Functional Health Literacy was used to measure literacy.

**Results:**

Of 172 subjects with HF and diabetes, 27% had limited literacy compared to 15% of 826 subjects without HF (OR 2.05; 95% CI 1.39, 3.02; *P *< 0.001). Adjusting for age, sex, race, income, marital status and health insurance, HF continued to be significantly associated with limited literacy (OR 1.55, 95% CI 1.00, 2.41, *P *= .05).

After adjusting for education, however, HF was no longer independently associated with literacy (OR 1.31; 95% CI 0.82 – 2.08; *P *= 0.26).

**Conclusion:**

Over one quarter of diabetic adults with HF have limited literacy. Although this association is no longer statistically significant when adjusted for education, clinicians should be aware that many of their patients have important limitations in dealing with written materials.

## Background

The 2003 National Assessment of Adult Literacy estimated that 30 million Americans are at Below Basic literacy level and are able to do only simple and concrete literacy tasks. Another 63 million are at the Basic literacy level and are only able to read and understand short and commonplace texts [1]. Health Literacy was measured as a new and separate component in the 2003 National Assessment of Adult Literacy where 36% percent scored at the Below Basic or Basic health literacy level. Among adults 65 and older, 59% were at the Below Basic or Basic levels of health literacy [2].

As defined by the National Institutes of Health, health literacy is the degree to which individuals have the capacity to obtain, process, and understand basic health information and services needed to make appropriate health decisions [3]. Health literacy encompasses more than just the ability to read written materials. It also means understanding the information in order to actively participate in managing health. Even people with average or strong reading ability could be at risk for low health literacy because they may find medical terminology and medical concepts foreign. Williams *et al*. [4] reported that over one third of the patients evaluated in two public hospitals were unable to read and comprehend basic health-related teaching materials.

A systematic review of U.S. studies concluded that nearly half of the total subjects had limited health literacy [5]. Another study examined Medicare enrollees (many with chronic conditions including heart failure), and found that more than one third of the respondents had limited literacy [6].

Evidence continues to emerge about the adverse influence of limited health literacy on health outcomes. Limited literacy is associated with decreased knowledge of one's medical condition [7-12], non-adherence to treatment plans [9], poor self care behaviors [7,8,13,14], and increased hospitalizations [15,16]. Dewalt's systematic review concluded that people who read at low levels are 1.5 to 3 times more likely to have an adverse outcome compared to people who read at higher levels [17].

Limited health literacy is associated with education [6,9,12,18,19], age [4,6-8,12], ethnicity [6,9,11,12], income [6,9], and chronic diseases including diabetes, AIDS, hypertension and asthma [6-9,14,20]. Aged, minority, poor, and medically complex individuals are uniquely vulnerable in our convoluted health care systems and may be even more so if they are functionally low literate.

Little is known about the specific prevalence of limited literacy among adults with HF. Using a convenience sample of adults with diabetes we sought to determine the prevalence of limited literacy in diabetic patients with HF compared to those with diabetes and no HF. We also sought to determine if clinically relevant variables shown to be related to limited literacy in other studies, (age, sex, race, marital status, insurance, education, and income) impact literacy.

## Methods

### Setting and Study Participants

This study was part of a larger project, the Vermont Diabetes Information System (VDIS). There are 73 practices, 147 primary care providers, and 8,880 patients in the VDIS registry [21]. The providers are all non-academic Family Medicine or General Internal Medicine physicians, nurse practitioners or physician assistants working in solo practices or small-to-medium sized groups in northern New York, Vermont, or northern New Hampshire. The patients include all the adults with diabetes seen in each practice.

Patient names were reviewed by the provider to confirm the diagnosis of diabetes and to exclude patients with significant cognitive impairment. Random patients were then contacted by telephone until a sample of approximately 15% of the patients from each practice agreed to participate in an interview. Of the 1,576 patients we attempted to contact, 36% (570) were not reached or declined. The 7,801 non-interviewed subjects of VDIS, (which includes those who declined and those never contacted) were younger (mean age of 63 versus 65, *P *< 0.001); more likely to be men (49% versus 46%; *P *= 0.06) than those who completed the interview. Reasons patients declined to participate was not collected.

Demographic information including age, sex, race, education, income, health insurance, and marital status, as well as self-reported data on comorbidities, were obtained by questionnaire mailed to participants prior to the home interview.

Participants were identified as having HF if they checked "yes" on the co-morbidity question of the Self-Administered Comorbidity Questionnaire: Do you have or have you had heart failure? (You may have been short of breath and your doctor may have told you that you had fluid in your lungs or that your heart was not pumping well.)

Interviews occurred between July 2003 and March 2005. At the time of the home interview, a research assistant reviewed the questionnaire for completeness and administered the Short Test of Functional Health Literacy for Adults. The research assistant read each item on the questionnaire aloud that the subject could not read or did not complete before the interview. Subjects with low vision or other disabilities were read the entire survey. Patients received a twenty dollar honorarium after completion of the home interview. The University of Vermont Committee on Human Research in the Medical Sciences approved the protocol for this study and all subjects gave written informed consent to participate in the interview.

### Measures

We used the Short Test of Functional Health Literacy in Adults (STOFHLA) to measure literacy. The STOFHLA is a 36-item, 7-minute timed test of reading comprehension that employs the Cloze procedure, in which a word in a sentence is omitted and must be chosen from a multiple choice list. The STOFHLA uses passages from instructions for preparation for an upper gastrointestinal series and the "Rights and Responsibilities" section of a Medicaid application. Results are categorized into inadequate (0–16 correct answers), marginal (17–22), and adequate health literacy (23–36). The STOFHLA has demonstrated good internal consistency (Cronbach's alpha = 0.98 for all items combined) and concurrent validity compared to the long version, the TOFHLA (r = 0.91) [22].

Comorbidity was measured with the Self-Administered Comorbidity Questionnaire (SCQ) [23], a modification of the widely used Charlson Index. It uses patient interview or questionnaire rather than chart abstraction for assessment of comorbidity and has excellent agreement with the chart-based Charlson Index. The SCQ asks about existence of a health condition, the severity of the condition, and limitations in function associated with the condition. The question: "Do you have or have you had any of the following problems?" was asked in relation to 18 conditions including heart attack, heart failure, claudication ("blockages in the arteries in your legs"), stroke, kidney disease, and others. For each problem, the subject is asked "Do you receive treatment for it?" and "Does it limit your activities?" An individual can score up to 3 points for each medical condition: 1 point for the presence of the problem, another point for treatment, and an additional point for limitation in functioning. All patients had diabetes and no additional points were assigned for its presence. The maximum possible score was 54 points.

Sangha and colleagues reported the test-retest reliability for the SCQ as 0.94 by the intraclass correlation coefficient and 0.81 by the Spearman correlation coefficient. The Spearman correlation between the SCQ and the Charlson Index was 0.32 for the whole instrument and 0.55 for truncated versions. Overall agreement exceeded 90% except for lung disease (78%) and heart disease (88%) [23].

### Analysis

Using the STOFHLA score as a continuous variable, we drew box plots to illustrate variation in literacy between those with and without HF. We combined the inadequate and marginal literacy groups because they both need alternatives to typical written material. We then analyzed literacy as a dichotomous variable (limited literacy [STOFHLA score 0–22] *vs*. adequate literacy [STOFHLA score 23–36]). T-tests and Chi-square tests were used to analyze differences in patient characteristics by heart failure status.

We used unadjusted logistic regression to examine the association between the predictor, heart failure, and the outcome, limited literacy. We then performed multivariate logistic regression to examine the association between heart failure and literacy after controlling for clinically relevant variables and those shown to be related to limited literacy in other studies: age, sex, race, marital status, insurance, and income. Because the association between education and literacy is already well known [24], we performed the analysis again with all the covariates as well as education. The analysis was performed again excluding subjects with self reported poor vision or other physical impairment as is often done in studies assessing health literacy. All analyses were performed with STATA 8.2 (StataCorp, College Station, Texas).

## Results

**Figure 1:**
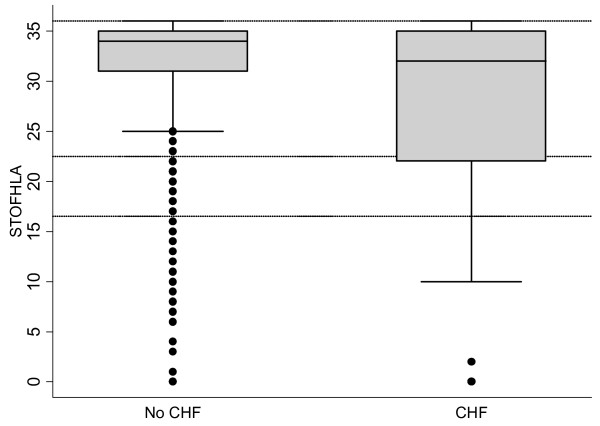
Comparison of Literacy in 998 Adults with Diabetes with and without Heart Failure (CHF).

Nine hundred and ninety eight subjects provided complete data for literacy and heart failure. The mean age was 65 years and just over half were women. Most were white, had at least a high school education and had health insurance. Seventeen percent had limited literacy (STOFHLA < 22) (Table 1). Out of the 172 who reported heart failure, 137 (80%) reported currently receiving treatment for their heart failure, and 100 (58%) reported that their heart failure limits their activities. Compared to subjects without HF, those with HF were more likely to be male, older, less educated, and poorer. Twenty-seven percent of the subjects with both diabetes and HF had limited literacy, compared to 15% of the subjects who had diabetes but no HF (*P *< 0.001) (Table 1). A box plot (Figure 1) illustrates the variation in literacy between those with and without HF.

**Table 1:** Characteristics of 998 adults with diabetes with and without heart failure (HF)

*Characteristic*	*All Subjects (n = 998)*	*Subjects with HF (n = 172)*	*Subjects without HF (n = 826)*	*P*
Age, years, mean (range)	65 (22–93)	69 (45–90)	64 (22–93)	<0.001
Age ≥ 65 years, n (%)	524 (53)	114 (66)	410 (50)	<0.001
Female, n (%)	543 (54)	84 (49)	459 (56)	0.11
White race, n (%)	968 (97)	165 (96)	803 (97)	0.48
Married or living as married, n (%)	623 (63)	96 (56)	527 (64)	0.06
Annual income < $30,000, n (%)	543 (59)	120 (75)	423 (56)	<0.001
High School Graduate, n (%)	746 (75)	102 (60)	644 (78)	<0.001
No health insurance, n (%)	24 (2)	3 (2)	21 (3)	0.54
Limited Literacy, n (%)	171 (17)	46 (27)	125 (15)	<0.001
Adequate Literacy, n (%)	827 (83)	126 (73)	701 (85)	<0.001

Limited Literacy includes those with Short Test of Functional Health Literacy Score < 23, blind or otherwise unable to complete the test.

In unadjusted analyses, there was a significant relationship between HF and literacy (OR 2.05; 95% CI 1.39, 3.02; *P *< 0.001). Nine hundred and nine patients provided complete data for the multivariate analyses. After adjusting for age, sex, race, income, marital status, and health insurance, HF was still significantly associated with limited literacy (OR 1.55, 95% CI 1.00, 2.41, *P *= .05). When education was added to the model, the relationship between HF and literacy was no longer significant (OR 1.31; 95% CI 0.82 – 2.08; *P *= 0.26). Excluding the 27 subjects who reported they had poor vision or other physical impairments had little effect on the analysis. In all the analyses, limited literacy was significantly associated with older age, male sex, and low income, but not with race, marital status or health insurance (Table 2).

**Table 2:** Adjusted odds of limited literacy for 909 adults with diabetes

	*Adjusted for social and economic factors*		*Adjusted for all covariates*	
*Characteristic*	*Odds Ratio (CI)*	*P*	*Odds Ratio (CI)*	*P*
Heart Failure	1.55 (1.00, 2.41)	0.05	1.31 (0.82–2.08)	0.26
Age ≥ 65 years	3.45 (2.19, 5.44)	<0.001	3.51 (2.18–5.63)	<0.001
Male sex	1.77 (1.17, 2.66)	0.01	1.60 (1.04–2.46)	0.03
White race	0.48 (0.16, 1.41)	0.18	0.45 (0.14–1.43)	0.18
<High School Education			5.04 (3.31–7.69)	<0.001
Income < $30,000 per year	5.17 (2.98, 8.95)	<0.001	2.85 (1.59–5.13)	<0.001
Married or living as married	1.00 (0.66, 1.52)	0.98	0.94 (0.61–1.46)	0.79
No health insurance	0.83 (0.18–3.85)	0.81	0.75 (0.15–3.71)	0.72

CI = 95% confidence interval. Limited Literacy includes those with Short Test of Functional Health Literacy Score < 23, blind or otherwise unable to complete the test.

## Discussion

Unadjusted, the prevalence of limited literacy among adults with diabetes and HF is nearly twice as high (27%) as those with diabetes who do not have HF (15%). The overall prevalence of limited literacy in this population is on the low end of the reported range in a systematic literature review that found that 25–50% of adults in outpatient medical settings had limited literacy [5]. Gazmararian and colleagues reported limited literacy in 27% to 44% of new Medicare enrollees in four large US cities in 1999 [6]. In a later study, the same group reported limited literacy in 36% of Medicare enrollees with chronic disease (116 had HF) [25]. The lower prevalence reported here may reflect that this population is younger and less likely to be foreign born.

The relationship between literacy and HF was somewhat weaker but still statistically significant after adjusting for likely confounders such as age, sex, race, income, marital status and insurance (but not education). However, it became weaker still and lost statistical significance when controlling for education as well. This suggests that education is either in the causal chain or is a confounder with a direct relationship to both low literacy and heart failure. We find it implausible that heart failure directly causes low educational status, or that low educational status causes heart failure (at least directly). However, the data are consistent with a possible mediating role for literacy in the relationship between education and heart failure [26]. In any event, adjusting for education may obscure the fact that, causal or not, literacy is highly associated with heart failure. One in four subjects with HF in this study may require alternatives to written materials to assist them in understanding their disease and following through with self-care. Although limited literacy may or may not be directly caused by HF *per se*, the high prevalence in this cohort warrants consideration when working with this population.

These data support previous reports of limited literacy among the elderly [5,6]. Older adults have a large burden of chronic diseases and thus considerable health related reading demands [6]. Given that the incidence of HF increases with age [27], and with the expanding group of older adults in America, the association of limited literacy and age becomes problematic from a clinical and practical point of view.

Many HF patients lack sufficient knowledge to manage their disease, highlighting the need for improved patient education [28,29]. Patients with limited literacy are significantly less likely to correctly answer questions about HF than those with adequate literacy [25]. In addition, a number of investigators have reported deficient heart failure self-care behaviors among patients despite HF education [30,31]. The wide gap between receiving information that is presented and acting on it may be the result of low literacy. Limited literacy is likely to influence the learning of essential HF self-care skills leading to poorer health outcomes.

There are limitations to this study. The subjects all had diabetes, were enrolled in primary care, were primarily white, and lived in Vermont and bordering states in a mostly rural environment limiting generalizability. Subjects self reported whether or not they had HF and their response was not verified by their providers. We excluded subjects with significant cognitive impairment based upon assessment by their primary care provider. In addition, our study may be influenced by participation bias: individuals with limited literacy may have been more likely to decline to participate in the survey. We don't have any information on the 570 patients who declined to be interviewed other than many of them are younger and we surmise that their time commitment with work and other responsibilities may have limited their participation. The cross sectional design of the study tells us about associations and not causality and reflects the status of literacy at only one point in time. Additional studies are needed to determine the prevalence of limited literacy in adults with HF without diabetes.

## Conclusion

Twenty-seven percent of the adults in this study who have 2 chronic health conditions, diabetes and HF, have limited literacy compared to 15% of the adults with diabetes that do not have HF. After adjusting for age, race, sex, income, insurance, and marital status (but not education), there continues to be a significant association between HF and limited literacy. The relationship of literacy and HF appears to be confounded or mediated by education. Health care providers need to be aware that adults with both diabetes and HF are at increased risk for having limited literacy. Understanding the extent of limited literacy among adults with chronic diseases can lead to enhanced efforts to improve patients knowledge and skills and ultimately, may improve the outcomes of their disease management.

## Competing interests

The author(s) declare that they have no competing interests.

## Authors' contributions

ASL conceived of the study, participated in the coordination, interpretation of the data, and drafted the manuscript. NM, BN participated in its design, performed statistical analyses, and interpreted the data. All authors reviewed the manuscript for important intellectual content and gave final approval.

## Pre-publication history

The pre-publication history for this paper can be accessed here:


